# Procuring Abortion

**Published:** 1850-01

**Authors:** 


*Procuring Abortion.* [*Extracted from a manuscript work by Enos Stevens, Examining Agent for the Massachusetts Commissioners for the Prevention and Restoration from Idiocy.*]—Among the 240 Idiots described by the Commissioners to the Legislature of Massachusetts, seven seem to have been made so by their mothers trying to procure abortion by using very powerful drugs. Although these unborn children were not thus quite killed, yet they were irrecoverably stupefied and malformed to the lowest degree of both mental and animal idiocy and weakness. Indeed these children still remain glaring, crawling and howling personifications of crime, misery, and a long continued corruption and death. In some of these cases the health of the women was ruined for the remainder of their lives, and they ever after continued to bring forth idiots, malformations and invalids. Yet one woman, the mother of the very lowest of these seven idiots, communicated all her drugs to her unwedlocked child, so that her subsequent children are all living, well and intelligent.—*Boston Med. and Surg. Journal.*

